# Microglial TREM2/DAP12 Signaling: A Double-Edged Sword in Neural Diseases

**DOI:** 10.3389/fncel.2018.00206

**Published:** 2018-08-06

**Authors:** Hiroyuki Konishi, Hiroshi Kiyama

**Affiliations:** Department of Functional Anatomy and Neuroscience, Nagoya University Graduate School of Medicine, Nagoya University, Nagoya, Japan

**Keywords:** damage, degeneration, inflammation, injury, ITAM, regeneration, Syk, TYROBP

## Abstract

Microglia are activated after neuronal injury and in neurodegenerative diseases, and trigger neuroinflammation in the central nervous system (CNS). Microglia-derived neuroinflammation has both beneficial and detrimental effects on neurons. Because the timing and magnitude of microglial activation is thought to be a critical determinant of neuronal fate, understanding the molecular mechanisms underlying microglial activation is required to enable establishment of microglia-targeted therapies for neural diseases. Plasma membrane receptors play primary roles as activators of microglia and in this review, we focus on a receptor complex involving triggering receptor expressed on myeloid cells 2 (TREM2) and DNAX-activating protein of 12 kDa (DAP12), both of which are causative genes for Nasu-Hakola disease, a dementia with bone cysts. Recent transcriptome approaches demonstrated TREM2/DAP12 signaling as the principal regulator that transforms microglia from a homeostatic to a neural disease-associated state. Furthermore, animal model studies revealed critical roles for TREM2/DAP12 in the regulation of microglial activity, including survival, phagocytosis, and cytokine production, not only in Alzheimer's disease but also in other neural diseases, such as Parkinson's disease, demyelinating disease, ischemia, and peripheral nerve injury. Intriguingly, while TREM2/DAP12-mediated microglial activation is detrimental for some diseases, including peripheral nerve injury, it is beneficial for other diseases. As the role of activated microglia differs among disease models, TREM2/DAP12 signaling may result in different outcomes in different diseases. In this review we discuss recent perspectives on the role of TREM2/DAP12 in microglia and their contribution to neural diseases.

## Introduction

Microglia are macrophage-like myeloid cells in the central nervous system (CNS). Besides macrophage-related immunological functions, microglia play CNS-specific roles, such as maintenance of brain homeostasis and modulation of neural circuits (Wake et al., 2009; Paolicelli et al., 2011; Parkhurst et al., 2013; Ueno et al., 2013). Microglia exhibit a ramified morphology in the healthy adult brain and they survey their surrounding area with motile processes under physiological conditions (Davalos et al., 2005; Nimmerjahn et al., 2005; Hanisch and Kettenmann, 2007). However, upon neuronal injury and in neurodegenerative diseases, microglia become activated, and transform into a hypertrophic or ameboid shape (Lobsiger et al., 2013; Roth et al., 2014; Fernández-Arjona et al., 2017). Activated microglia are thought to have opposing functions (David and Kroner, 2011; Hu et al., 2015). They secrete a variety of neurotrophic factors to protect damaged neurons and phagocytose cellular debris to enable tissue regeneration (Nakajima and Kohsaka, 2004; Neumann et al., 2009); however, when microglia are excessively activated, they can damage neurons by secreting neurotoxic molecules, such as nitric oxide (NO) and reactive oxygen species (ROS), and eventually phagocytose injured neurons (Block et al., 2007; Brown and Neher, 2014; Fu et al., 2014). Thus, microglia may be critical determinants of neuronal fate and establishing a method to effectively control microglial activity would contribute to the treatment of neural diseases.

Receptors expressed on the microglial surface play roles in sensing environmental changes around microglia and regulate their activation (Kierdorf and Prinz, 2013). In this regard, recently there has been focus on a receptor complex, the triggering receptor expressed on myeloid cells 2 (TREM2), and the DNAX-activating protein of 12 kDa (DAP12) [also known as TYRO protein kinase-binding protein (TYROBP) or killer cell activating receptor-associated protein (KARAP)]. As TREM2 is a strong risk factor for Alzheimer's disease (AD) in humans and was recently demonstrated to be a critical regulator of microglial activity in mouse models of AD (Guerreiro et al., 2013; Jonsson et al., 2013; Jay et al., 2015; Wang et al., 2015; Keren-Shaul et al., 2017; Ulland et al., 2017), many articles have reviewed the recent progress of TREM2/DAP12 research in AD (Ma et al., 2015; Colonna and Wang, 2016; Mecca et al., 2018). However, TREM2/DAP12 have also been shown to control microglial activity and consequently affect the fate of damaged neurons after neuronal injury and in neurodegenerative diseases besides AD. In this review, we start with a brief introduction of the TREM2/DAP12 complex, shortly summarize the studies of TREM2/DAP12 in AD and then highlight the roles of TREM2/DAP12 in other animal models of neural disease.

## The TREM2/DAP12 complex

**Figure 1:**
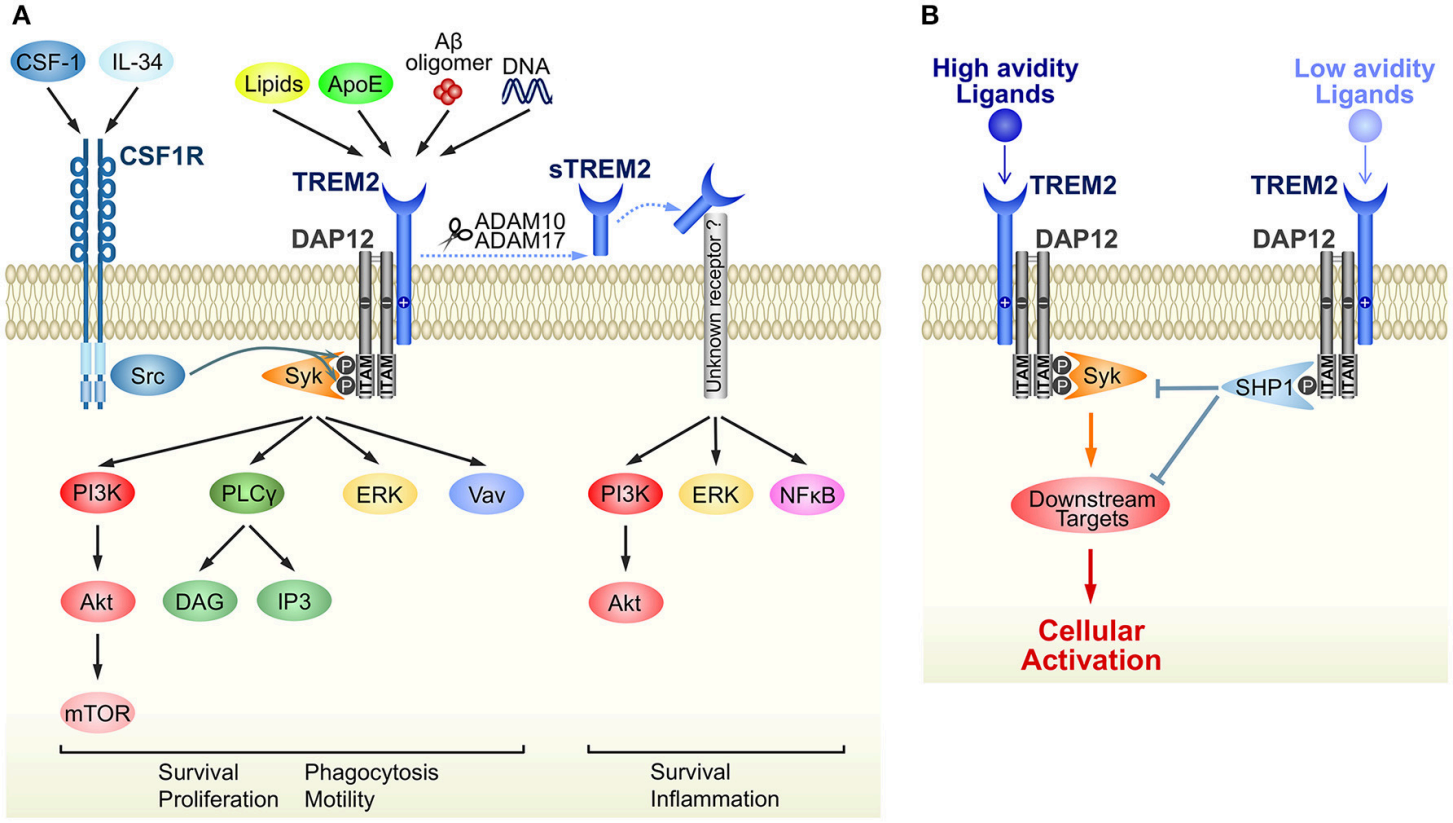
Schematic representation of TREM2/DAP12 signaling in microglia. **(A)** Ligands and downstream signaling of TREM2/DAP12. Of the known TREM2 ligands, only ligands that highly correlate with neural diseases are shown. Upon ligand binding to TREM2, two tyrosine residues within the ITAM motif of DAP12 are phosphorylated, which recruits Syk kinase to activate downstream signaling molecules, such as ERK, PI3K, PLCγ, and Vav. Src, the main effector of CSF1R, is a kinase supposed to phosphorylate the ITAM tyrosine residues. The soluble form of TREM2, sTREM2, is generated by ectodomain shedding by ADAM 10 or 17, which activates PI3K, ERK and NFκB via an unknown receptor. Note that parts of the signaling pathways are inferred from studies of other types of myeloid cells, such as macrophages and osteoclasts. **(B)** A putative mechanism by which TREM2/DAP12 generates opposing signals. Upon binding of high avidity ligands to TREM2, both tyrosine residues in the ITAM motif become phosphorylated and the recruited Syk kinase activates downstream signaling molecules, as shown in **(A)**. Conversely, in the case of low avidity ligands, only partial phosphorylation occurs and then activated SHP-1 phosphatase dephosphorylates molecules downstream of Syk signaling to inhibit cellular activation.

The immunoreceptor tyrosine-based activation (ITAM) motif in the intracellular domain of some transmembrane proteins generates an activation signal in leukocytes (Cambier, 1995). DAP12 was identified as an ITAM-containing, disulfide bond-linked homodimer expressed on natural killer cells (Lanier et al., 1998). Because of its short extracellular domain, DAP12 itself is thought to have no ligand-binding capability. Instead, DAP12 forms complexes with some ligand-binding receptors (DAP12-associated receptors) and transduces signals from DAP12-associated receptors into the cytoplasm (Figure 1A). TREM2 was initially identified as a TREM1 homolog in an expressed sequence tag database (Bouchon et al., 2000) and was later shown to bind to DAP12 via oppositely-charged residues in their transmembrane domains (Bouchon et al., 2001; Daws et al., 2001). Upon ligand binding to TREM2, tyrosine residues within ITAM are phosphorylated, recruiting Syk kinase to activate downstream signaling molecules such as extracellular signal-regulated protein kinase (ERK), phosphatidylinositol 3-kinase (PI3K), phospholipase Cγ (PLCγ), and Vav (Takahashi et al., 2005; Otero et al., 2009; Peng et al., 2010; Wang et al., 2015; Colonna and Wang, 2016) (Figure 1A). Downstream signals from DAP12 and colony-stimulating factor-1 receptor (CSF1R) show crosstalk; for example Src tyrosine kinase, the main effector of CSF1R signaling, phosphorylates the ITAM motif of DAP12 (Zou et al., 2008; Otero et al., 2009) (Figure 1A).

Although ITAM is generally considered a signaling motif leading to cellular activation, TREM2/DAP12 complex can also induce inhibitory signals (Hamerman et al., 2006; Turnbull et al., 2006). This enigmatic function is hypothesized to result from the ligand affinity/avidity of DAP12-associated receptors (Turnbull and Colonna, 2007) (Figure 1B). When a low affinity/avidity ligand binds to a DAP12-associated receptor, the ITAM motif of DAP12 becomes partially, not fully, phosphorylated. The partial phosphorylation of ITAM recruits the SH2 domain-containing protein tyrosine phosphatase SHP-1, leading to dephosphorylation of downstream targets of Syk kinase, and inhibition of cellular activation (Peng et al., 2010).

## TREM2/DAP12 in neural diseases

**Figure 2:**
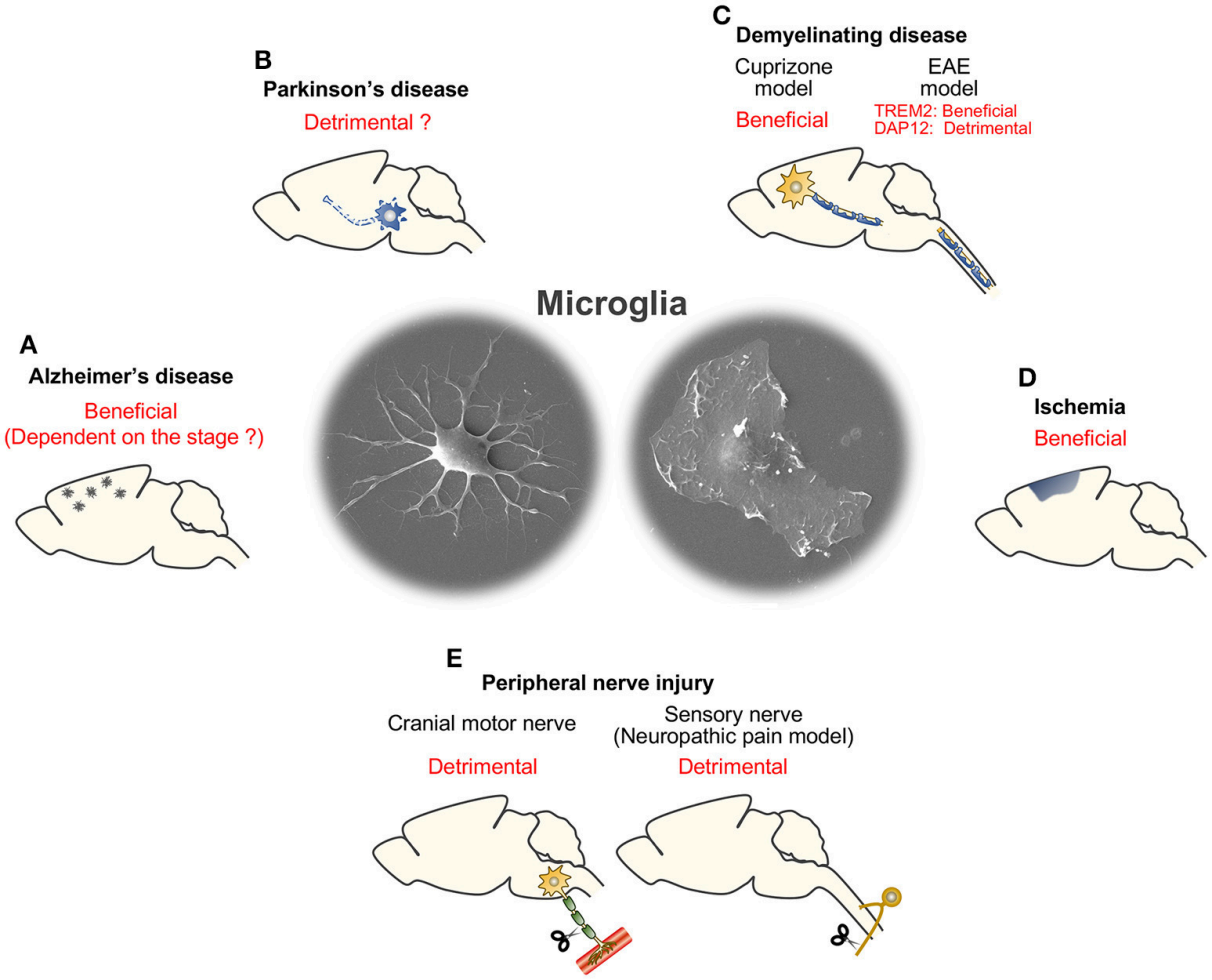
Summary of consequences of microglial TREM2/DAP12 in neural diseases. **(A)** Alzheimer's disease. **(B)** Parkinson's disease. **(C)** Demyelinating disease (cuprizone and EAE models). **(D)** Ischemia. **(E)** Peripheral nerve injury (cranial motor nerve injury and sensory nerve injury models).

The functions of TREM2/DAP12 in neural diseases are summarized in Table 1 (excluding AD) and Figure 2.

**Table 1:** Microglial TREM2/DAP12 function in neural diseases other than AD.

**Disease model**	**Manipulation tool**	**Phenotype (↑: Increase, ↓: Decrease, → : No change)**	**Effect on neuronal fate**	**References**
**PARKINSON'S DISEASE**				
MPP (Mix culture)	DAP12 loss-of-function mouse (KΔ75)	Loss of dopaminergic neuron ↓	DAP12: detrimental	Kinugawa et al., 2013
MPTP	DAP12 loss-of-function mouse (KΔ75)	Microglial number → Loss of dopaminergic neuron →	DAP12: No effect	
6-OHDA	DAP12 loss-of-function mouse (KΔ75)	Microglial number ↓ Loss of dopaminergic neuron ↓	DAP12: detrimental	Virgone-Carlotta et al., 2013
		Behavioral alteration ↓		
MPTP	TREM2 KO mouse	Microglial number ↓	TREM2: No effect	Belloli et al., 2017
		[^11^C]PK11195 uptake by microglia ↑		
		Pro-inflammatory molecule (IL-1β, TNF-α) ↓		
		Loss of DAT activity →		
MPTP	TREM2 overexpression by adenovirus	Microglial number ↓ Pro-inflammatory molecule (IL-1β, TNF-α, iNOS, COX-2) ↓	TREM2: beneficial	Ren et al., 2018
		Loss of TH immunoreactivity ↓		
**DEMYELINATING DISEASE**				
EAE	DAP12 loss-of-function mouse (KΔ75)	Activation marker of microglia (MHCII) ↓ Number of infiltrating leucocytes ↓	DAP12: detrimental	Bakker et al., 2000a
		EAE Severity (Clinical score) ↓		
EAE	Transplantation of TREM2-transduced bone marrow-derived myeloid cells	Number of phagocytic cells ↑ Clearance of myelin debris ↑ Pro-inflammatory molecule (IL-1β, TNF-α, IFN-γ) ↓ Anti-inflammatory molecule (IL-10) ↑ Tissue damage of spinal cord ↓	TREM2: beneficial	Takahashi et al., 2007
		EAE Severity (Clinical score) ↓		
EAE	TREM2 functional blocking antibody	Leucocytic infiltration ↑ Demyelination ↑ EAE Severity (Clinical score) ↑	TREM2: beneficial	Piccio et al., 2007
Cuprizone	TREM2 KO mouse	Microglial number ↓	TREM2: beneficial	Poliani et al., 2015
		Dystrophic morphology of microglia		
		Pro-inflammatory molecule (IL-1β, IL-6, etc.) ↓		
		Phagocytic molecule (Axl) ↓		
		Molecules for lipid transport and metabolism (ApoE etc.) ↓		
		Clearance of myelin debris ↓		
		Axonal damage ↑		
		Remyelination ↓		
Cuprizone	TREM2 KO mouse	Microglial number ↓	TREM2: beneficial	Cantoni et al., 2015
		Less activated morphology of microglia		
		Activation marker of microglia (Mac-3, MHCII) ↓		
		Pro-inflammatory molecule (iNOS) ↓		
		Molecules for lipid metabolism (Lipoprotein lipase) ↓		
		Clearance of myelin debris ↓		
		Axonal damage ↑		
		Neurological deficit ↑		
**ISCHEMIA**				
Transient middle cerebral artery occlusion	TREM2 KO mouse	Microglial number ↓ Pro-inflammatory molecule (IL-1α, IL-1β, TNF-α) ↓ Phagocytic marker (CD68) ↓ Infarct size →	TREM2: No effect	Sieber et al., 2013
Permanent middle cerebral artery occlusion	TREM2 KO mouse	Number of IB4^+^ activated microglia ↓ Number of CD68^+^ phagocytic microglia ↓ Contact of microglia with apoptotic cells ↓ Infarct size ↑ Neurological deficit ↑	TREM2: beneficial	Kawabori et al., 2015
Transient middle cerebral artery occlusion	TREM2 siRNA	Pro-inflammatory molecule (IL-1β, TNF-α) ↑ Anti-inflammatory molecule (IL-10) ↓ NF-κB phosphorylation ↑ Number of apoptotic neuron ↑ Infarct size ↑	TREM2: beneficial	Wu et al., 2017
Transient middle cerebral artery occlusion	TREM2 siRNA	Pro-inflammatory molecule (iNOS) ↑ Anti-inflammatory molecule (Arg-1) ↓	TREM2: beneficial	Zhai et al., 2017
		Number of apoptotic neuron ↑		
		Infarct size →		
		Neurological deficit →		
	TREM2 overexpression by lentivirus	Pro-inflammatory molecule (iNOS) ↓		
		Anti-inflammatory molecule (Arg-1) ↑		
		Number of apoptotic neuron ↓		
		Infarct size ↓		
		Neurological deficit ↓		
**PERIPHERAL NERVE INJURY**				
Motor nerve injury	DAP12 KO mouse	Microglial number ↓ Pro-inflammatory molecule (IL-1β, IL-6, TNF-α, IRF8, etc.) ↓ Neuronal damage ↓	DAP12: detrimental	Kobayashi et al., 2015
Neuropathic pain (Sensory nerve injury)	DAP12 KO mouse	Microglial proliferation → Activation marker of microglia (CD11b) ↓ Pain-related molecule (Cathepsin S, BDNF) ↓ Neuropathic pain ↓	DAP12: detrimental for pain	Guan et al., 2016
Neuropathic pain (Sensory nerve injury)	DAP12 KO mouse	Microglial number ↓ Pro-inflammatory molecule (IL-1β, IL-6, TNF-α, IRF8, etc) ↓ Pain-related molecule (Cathepsin S, P2RX4) ↓ Neuropathic pain ↓	TREM2/DAP12: detrimental for pain	Kobayashi et al., 2016
	TREM2 agonistic antibody	Pro-inflammatory molecule (IL-1β, TNF-α, IRF8) ↑		
		Neuropathic pain ↑		
Motor nerve injury	TREM2 KO mouse	Neuronal damage ↓	TREM2: detrimental	Krasemann et al., 2017
**Epilepsy**				
	TREM2 KO mouse	Microglial number ↓		Zheng et al., 2017
		Less activated morphology of microglia		
**Traumatic brain injury**				
	TREM2 KO mouse	Lesion site: Number of microglia/macrophages ↑ Away from lesion: Number of macrophages ↓ Away from lesion: Pro-inflammatory molecule (TNF-α) ↓ Away from lesion: Hippcampal damage ↓ Neurological deficit ↓	TREM2: detrimental	Saber et al., 2017
**Focal laser ijury**				
	TREM2 KO mouse	Speed of process extension ↓		Mazaheri et al., 2017

### TREM2/DAP12 in AD

After the discovery of the TREM2/DAP12 complex in the immune system, both molecules were sequentially identified as causative genes for Nasu-Hakola disease [also known as polycystic lipomembranous osteodysplasia with sclerosing leukoencephalopathy (PLOSL)], which is characterized by presenile dementia with bone cysts (Paloneva et al., 2000, 2002). Because TREM2 and DAP12 are predominantly expressed by microglia (Bakker et al., 2000b; Paloneva et al., 2001; Schmid et al., 2002), microglial dysfunction caused by TREM2/DAP12 impairment was assumed to be involved in the pathogenesis of Nasu-Hakola disease (Paloneva et al., 2000, 2001; Schmid et al., 2002). Almost a decade later, a breakthrough was made by studies demonstrating that a rare variant of TREM2 (loss-of-function mutation, R47H) is a risk factor for AD and frontotemporal dementia (Guerreiro et al., 2013; Jonsson et al., 2013). In the same period, DAP12 was also reported as a key regulator of late-onset AD by an integrative network-based approach (Zhang et al., 2013). AD model mice crossed with *Trem2*- or *Dap12*-deficient strains revealed that the TREM2/DAP12 complex is involved in microglial activation around amyloid plaques to prevent accumulation and diffusion of β-amyloid (Aβ) (Wang et al., 2015, 2016; Yuan et al., 2016; Keren-Shaul et al., 2017). TREM2/DAP12-mediated signals activate mammalian target of rapamycin (mTOR) to support microglial biosynthetic metabolism; *Trem2* deficiency impairs cellular metabolism and promotes increased autophagy in microglia in an AD mouse model (Ulland et al., 2017). Transgenic overexpression of human *TREM2* modified the morphological and functional responses of microglia, which resulted in amelioration of the pathology and memory deficits in an AD mouse model (Lee et al., 2018). Although overall, TREM2/DAP12-dependent cellular activation appears to be beneficial, Jay et al. suggested the possibility that the functional consequence of TREM2/DAP12 signaling depends on the stage of AD, with detrimental effects at the early stage and beneficial effects at the late stage (Jay et al., 2015, 2017).

Regarding TREM2 ligands, a variety of molecules including bacterial components have been identified (Kober and Brett, 2017). Among the known ligands, lipids such as phospholipids and glycolipids, and DNA, in particular, are predicted to be related to the pathology of dementia, as these molecules are thought to be released from or exposed on damaged cells (Cannon et al., 2012; Kawabori et al., 2015; Poliani et al., 2015; Wang et al., 2015). Apolipoprotein E (ApoE) is also considered an AD-associated ligand of TREM2 (Atagi et al., 2015; Bailey et al., 2015; Yeh et al., 2016) because the *APOE* genotype is a strong risk factor for late-onset AD (Corder et al., 1993). Very recently, Aβ oligomers were reported to bind directly to TREM2 and induce microglial activation with increased expression of pro-inflammatory cytokines (Zhao et al., 2018). Therefore, TREM2 recognizes multiple ligands in the extracellular milieu of the AD brain and generates intracellular activation signals via its signal transduction partner, DAP12.

In addition to its role as a receptor, the ectodomain of TREM2 can itself activate microglia (Figure 1A). As is the case with some receptor molecules, the ectodomain of TREM2 undergoes shedding by proteases in the “a disintegrin and metalloproteinase” (ADAM) family, including ADAM10 and ADAM17 (Kleinberger et al., 2014; Feuerbach et al., 2017; Schlepckow et al., 2017). The resulting soluble TREM2 (sTREM2) promotes survival and pro-inflammatory responses of microglia via the PI3K and nuclear factor (NF)-κB pathway, respectively (Zhong et al., 2017a). Another possible function of sTREM2 is as an inhibitor of TREM2; sTREM2 acts as a decoy receptor that competitively binds to TREM2 ligands (Piccio et al., 2008; Zhong et al., 2017a). As the concentration of sTREM2 in cerebrospinal fluid increases in AD patients, sTREM2 could be a potential biomarker for AD (Heslegrave et al., 2016; Piccio et al., 2016; Suárez-Calvet et al., 2016).

### Parkinson's disease (PD)

In addition to Alzheimer's disease, R47H and other variants of TREM2 are reported to be risk factors for PD (Benitez et al., 2013; Rayaprolu et al., 2013; Liu et al., 2016), although this remains controversial (Jonsson and Stefansson, 2013; Lill et al., 2015). Neuroinflammation caused by activated microglia is assumed to be detrimental in PD pathology. Activated microglia in the substantia nigra of the PD brain proliferate and produce neurotoxic molecules, such as NO, ROS and pro-inflammatory cytokines, resulting in progressive degeneration of dopaminergic neurons in a non-cell autonomous manner (Wu et al., 2003; Hu et al., 2008). Therefore, suppression of microgliosis and microglia-derived neurotoxicity is expected to be a therapeutic strategy for PD (Subramaniam and Federoff, 2017).

DAP12 function in a mouse model of PD was studied by two groups using KΔ75 knock-in mice, in which downstream signal transduction of DAP12 is impaired because of deletion of the cytoplasmic domain including the second ITAM motif (Tomasello et al., 2000). In the 6-hydroxydopamine (6-OHDA) model of PD in KΔ75 mice, microgliosis and neurodegeneration were suppressed (Virgone-Carlotta et al., 2013). The 1-methyl-4-phenylpyridinium (MPP)/1-methyl-4-phenyl-1,2,3,6-tetrahydropyridine (MPTP) model also revealed reduced *in vitro* neurotoxicity of microglia derived from KΔ75 mice; however, no alteration of neuropathology in KΔ75 mice was observed *in vivo* (Kinugawa et al., 2013). Although the *in vivo* consequence was different, both studies support the possibility that microglial neurotoxicity is suppressed in *Dap12*-deficient microglia. This is reminiscent of the finding that developmental apoptosis of hippocampal neurons is suppressed, possibly due to reduced neurotoxicity of KΔ75 microglia with lower levels of, for example, ROS production (Wakselman et al., 2008). The *Trem2* knockout (KO) mouse also demonstrated that *Trem2* deficiency results in reduced microglial numbers and decreased expression of pro-inflammatory cytokines, although the attenuated inflammation did not affect neuronal fate in the MPTP model (Belloli et al., 2017). Taken together, a TREM2/DAP12 signal likely promotes activation of microglia and subsequent neuroinflammation in the PD brain, occasionally leading to degeneration of dopaminergic neurons. However, a very recent study reported a conflicting result (Ren et al., 2018). The authors overexpressed *Trem2* using an adenoviral vector and showed that TREM2 overexpression attenuates pro-inflammatory responses of microglia and protects dopaminergic neurons from damage in the MPTP mouse model. In this study, TREM2 was presumably also transduced in cell types other than microglia and the ectopic expression of TREM2 may cause different results.

### Amyotrophic lateral sclerosis (ALS)

Shortly after the identification of the TREM2 variant R47H as a risk factor for AD, it was also reported to be a risk factor for sporadic ALS (Cady et al., 2014), although some studies showed no correlation (Rayaprolu et al., 2013; Lill et al., 2015). While TREM2 as a risk factor for ALS is under debate, the concentration of sTREM2 protein is higher in the cerebrospinal fluid of ALS patients, and *TREM2* was shown to be a member of the immune network module of ALS (Cooper-Knock et al., 2017). Given that microglia-derived neuroinflammation is involved in ALS pathology (Geloso et al., 2017; Liu and Wang, 2017), TREM2 is thought to play a role in the regulation of microglial activity in ALS. Although the ALS pathology of *Trem2-* or *Dap12*-deficient mice remains unknown, the molecular expression profile of microglia is significantly changed in *Trem2* KO mice with the superoxide dismutase 1 (SOD1)^G93A^ mutation (Krasemann et al., 2017). In the SOD1^G93A^ mouse model, the gene expression pattern of spinal microglia is altered, with downregulation of homeostatic genes and upregulation of inflammatory genes (Keren-Shaul et al., 2017; Krasemann et al., 2017). However, these alterations in gene expression are suppressed in *Trem2*-deficient microglia, suggesting that TREM2 is a key switch that transforms microglia from homeostatic to an ALS-associated phenotype (Krasemann et al., 2017).

### Demyelinating disease

Demyelination is one of the hallmarks of Nasu-Hakola disease (Verloes et al., 1997; Kobayashi et al., 2000). Adult *Dap12* KO and *Dap12* loss-of-function KΔ75 mice show reduced myelin in the brain, likely due to impaired myelination rather than demyelination (Kaifu et al., 2003; Nataf et al., 2005). Kaifu et al. found ectopic oligodendrocytes with aberrant differentiation near the hypomyelinated area in *Dap12* KO mice. They showed DAP12 expression in cultured oligodendrocytes in addition to microglia, raising the possibility that the hypomyelination might be a cell-autonomous phenotype of *Dap12*-deficient oligodendrocytes. However, given that DAP12 is predominantly expressed in microglia *in vivo* (Roumier et al., 2004; Thrash et al., 2009; Kobayashi et al., 2016), microglial dysfunction is suggested to be the primary cause of the hypomyelination phenotype observed in *Dap12*-deficient mice (Nataf et al., 2005). Some studies have demonstrated that microglia promote oligodendrocyte differentiation during remyelination after demyelination in the adult, as well as myelinogenesis in the neonate (Miron et al., 2013; Wlodarczyk et al., 2017). Both *Dap12* KO and KΔ75 mice have fewer microglia, with aberrant morphology, although the degree differs between the two strains (Nataf et al., 2005; Otero et al., 2009). This loss of functional microglia is suggested to lead to impaired differentiation of oligodendrocytes in *Dap12*-deficient mice.

In contrast to the two strains of *Dap12*-deficient mice described above, *Trem2* KO mice show no spontaneous abnormalities in myelin, even in aged mice, although in aged *Trem2* KO mice, the microglial number is smaller than age-matched wild-type (WT) mice and microglial morphology is dystrophic in restricted regions of the brain, including the corpus callosum (Poliani et al., 2015). However, after cuprizone-induced demyelination, myelin debris accumulated and axonal damage was augmented in *Trem2* KO mice (Cantoni et al., 2015; Poliani et al., 2015). Under demyelinating conditions, myelin lipids trigger TREM2 to induce microglial activation, including upregulation of some genes involved in pro-inflammatory responses and myelin clearance (Cantoni et al., 2015; Poliani et al., 2015). Thus, *Trem2*-deficient microglia show a less-activated or dystrophic morphology, decreased proliferative activity, and dysfunction of myelin clearance (Cantoni et al., 2015; Poliani et al., 2015). As a TREM2-mediated signal contributes to reprogramming of microglia for appropriate myelin clearance, which is thought to be required for remyelination (Lampron et al., 2015), TREM2 is a neuroprotective molecule in the cuprizone model.

In another model of demyelination, experimental autoimmune encephalomyelitis (EAE), excessive activation of microglia is thought to be detrimental for regeneration (Chu et al., 2018). Two types of study have addressed the role of TREM2 in EAE. Inflammation was promoted and the EAE clinical score and demyelination were worsened by intraperitoneal injection of a TREM2 functional blocking antibody (Piccio et al., 2007). Other studies transplanted TREM2-overexpressing bone marrow-derived myeloid cells (in which phagocytic activity was enhanced and pro-inflammatory gene expression was downregulated upon TREM2 stimulation) intravenously into mice with EAE (Takahashi et al., 2005, 2007). The transplanted TREM2-transduced myeloid cells successfully migrated to EAE lesions. The removal of degenerated myelin was promoted and expression of pro-inflammatory molecules such as *Il1b, Tnfa*, and *Ifng* [encoding interleukin (IL)-1β, tumor necrosis factor (TNF)-α, and interferon (IFN)-γ, respectively] was suppressed, resulting in reduced damage of not only remaining myelin but also axons. Although this transplantation study targeted bone marrow-derived myeloid cells and not microglia, taken together these studies indicate that the TREM2-mediated signal reduces neurotoxicity by suppressing pro-inflammatory responses in an EAE model. However, an apparently controversial result was obtained in *Dap12* KO mice with EAE (Bakker et al., 2000a). *Dap12* KO mice were shown to be highly resistant to EAE; disease onset was delayed and the clinical score was significantly better than WT mice. Thus, DAP12 is a neurotoxic molecule in the EAE model, while TREM2 is neuroprotective as described above. One possible reason for this discrepancy may be the variety of DAP12-associated receptors. As described in a later section, sialic acid-binding immunoglobulin-like lectin H (Siglec-H) is expressed by microglia as a DAP12-associated receptor, and signals derived from Siglec-H/DAP12 and TREM2/DAP12 complexes may oppositely regulate microglial activity in a mouse model of sensory nerve injury (Konishi et al., 2017). In addition to microglia, dendritic cells, which play a pivotal role as antigen-presenting cells in EAE pathogenesis (Mohammad et al., 2012), also express several DAP12-associated receptors besides TREM2 (Lanier, 2009). As TREM2 is one of the DAP12-associated receptors, the activation state of microglia or dendritic cells may be different between *Trem2-* and *Dap12*-deficient strains. Although the functional consequences of TREM2/DAP12 signaling in the EAE model are enigmatic, the role of TREM2/DAP12 may involve modulation of toll-like receptor signaling in microglia and/or dendritic cells (Ito and Hamerman, 2012; Kobayashi et al., 2015; Zhong et al., 2017b) because EAE development is dependent on toll-like receptors (Marta, 2009; Miranda-Hernandez and Baxter, 2013).

### Ischemia

Recent studies have revealed that microglial activation is spatiotemporally regulated and activated microglia play a biphasic role (Guruswamy and ElAli, 2017; Ma et al., 2017). Microglia are thought to enhance inflammation by releasing pro-inflammatory molecules and recruiting leukocytes in the acute phase. However, in the recovery phase, microglia may secrete anti-inflammatory cytokines to attenuate the inflammation and promote tissue repair.

Several reports analyzed TREM2 function in the mouse middle cerebral artery occlusion model of ischemia, in which the microglial number significantly increases in the ischemic hemisphere. Two different groups revealed that the increase in microglial number after ischemia was suppressed in *Trem2* KO mice (Sieber et al., 2013; Kawabori et al., 2015). *Trem2*-deficient microglia exhibited decreased expression of the phagocytic marker CD68 and reduced association with apoptotic cells (Sieber et al., 2013; Kawabori et al., 2015). The attenuated proliferation and phagocytic activity of *Trem2*-deficient microglia is consistent with the demyelination model induced by cuprizone described above (Cantoni et al., 2015; Poliani et al., 2015). Clearance of cellular debris is thought to be required for tissue reconstruction after injury (Neumann et al., 2009), and Kawabori et al. demonstrated a worsened neurological score in *Trem2* KO mice (Kawabori et al., 2015), suggesting a neuroprotective role for microglial TREM2 in ischemia. Regarding inflammatory responses of microglia, genes encoding pro-inflammatory cytokines such as IL-1β and TNF-α were downregulated in *Trem2* KO mice (Sieber et al., 2013), suggesting that TREM2 stimulates pro-inflammatory responses of microglia. In contrast, by overexpression or knockdown of *Trem2*, two other papers showed TREM2 is an inducer of anti-inflammatory responses (Wu et al., 2017; Zhai et al., 2017). The differences may arise from differences between the ischemic model or the timing of the analysis. However, it should be noted that TREM2 was consistently shown to be neuroprotective in all the studies (Kawabori et al., 2015; Wu et al., 2017; Zhai et al., 2017).

### Peripheral nerve injury

Motor nerve injury causes microglial activation around the cell bodies of injured motor neurons. Activated microglia change their shape to an amoeboid morphology and adhere to and enwrap cell bodies of injured neurons (Graeber et al., 1988). Although the function of activated microglia remains unclear, the adhesion or enwrapment is assumed to affect the fate of injured motor neurons (Yamada and Jinno, 2011). In the case of sensory nerve injury, in the peripheral branch of dorsal root ganglion neurons, for example, microglia become activated in the ipsilateral dorsal horn of the spinal cord. Activated microglia proliferate, secrete various pain-related molecules such as brain-derived neurotrophic factor (BDNF), and augment neuropathic pain (Inoue and Tsuda, 2018).

In a mouse model of motor nerve injury, microglial neurotoxicity was attenuated in both *Trem2* KO and *Dap12* KO mice (Kobayashi et al., 2015; Krasemann et al., 2017). TREM2 drives transformation of homeostatic microglia into a neurodegenerative phenotype, with upregulation of some inflammatory molecules (Krasemann et al., 2017). *Dap12* KO mice showed reduced microgliosis and lower expression of pro-inflammatory molecules (Kobayashi et al., 2015), which is reminiscent of the *Trem2* KO mice with cuprizone-induced demyelination described above (Cantoni et al., 2015; Poliani et al., 2015). Taken together, TREM2/DAP12-mediated microglial neuroinflammation enhances neurotoxicity to injured motor neurons (Kobayashi et al., 2015).

TREM2/DAP12 function was also addressed in a sensory nerve injury model (neuropathic pain model) (Guan et al., 2016; Kobayashi et al., 2016). Kobayashi et al. showed that microglial numbers as well as expression of pro-inflammatory molecules were decreased in the ipsilateral dorsal horn of *Dap12* KO mice compared with WT, leading to attenuated neuropathic pain. The study further revealed, using an agonistic antibody for TREM2, that TREM2 was the counterpart receptor of DAP12 in microglia involved in the DAP12-mediated inflammatory response (Kobayashi et al., 2016). Guan et al. also demonstrated that DAP12 leads to microglial activation as the downstream associated receptor of CSF1R after sensory nerve injury (Guan et al., 2016). *Dap12* KO mice demonstrated suppressed pain behavior and decreased expression of microglial pain-related genes (Guan et al., 2016), which is in line with the results reported by Kobayashi et al. (2016). However, the study showed normal proliferation of *Dap12*-deficient microglia in the dorsal horn (Guan et al., 2016), while Kobayashi et al. demonstrated fewer microglia in *Dap12* KO mice (Kobayashi et al., 2016). Although further confirmatory studies are needed for the DAP12-mediated proliferative activity of microglia, the TREM2/DAP12 complex, by cross-talking with CSF1R, would stimulate microglial activation after sensory nerve injury and exacerbate neuropathic pain (Inoue and Tsuda, 2018).

Amongst the known DAP12-associated receptors, Siglec-H is also expressed in microglia (Kobayashi et al., 2016; Konishi et al., 2017). A sensory nerve injury model showed enhanced pain behavior and increased expression of microglial pro-inflammatory cytokines in *Siglech* knockdown mice (Konishi et al., 2017). Thus, a Siglec-H/DAP12-mediated signal seems to act as a suppressor of microglial activation, which is in line with the anti-inflammatory role of Siglec-H in plasmacytoid dendritic cells in the immune system (Blasius et al., 2006; Takagi et al., 2011, 2016). By making a complex with either TREM2 or Siglec-H, DAP12 induces opposing signals in microglia in the injured dorsal horn. This paradoxical function of DAP12 is also suggested in the immune system (Blasius and Colonna, 2006; Turnbull and Colonna, 2007; Linnartz-Gerlach et al., 2014).

### Other injury models

In a kainic acid-induced epilepsy mouse model, microglia become activated and proliferate, particularly in the hippocampus (Zheng et al., 2011). *Trem2* KO mice had microglia with a less activated morphology (Zheng et al., 2017). The increase in microglial number after seizure was also suppressed in *Trem2* KO mice, which was suggested to result from both the attenuated proliferative activity and reduced viability of microglia in these mice. Other groups found downregulation of TREM2 expression in cortical tissues of patients with refractory epilepsy and in kainic acid-injected hippocampi in mice, which might be associated with alteration of microglial phagocytic activity in epileptic conditions (Abiega et al., 2016; Wyatt et al., 2017).

After traumatic brain injury, microglia, and infiltrating monocytes/macrophages trigger long-term neuroinflammation, which significantly affects the pathological, and functional outcome (Ziebell and Morganti-Kossmann, 2010). In the acute phase of traumatic brain injury, the numbers of microglia/macrophages are increased near the injury site in *Trem2* KO mice compared with WT (Saber et al., 2017). Conversely, the number of macrophages and expression of *Tnfa* are reduced at the periphery of the injury site in *Trem2* KO mice. The attenuated inflammatory response at the periphery of the injury results in reduced hippocampal atrophy and cognitive decline in *Trem2* KO mice. Thus, at least at the periphery of the lesion, TREM2 promotes pro-inflammatory responses that damage brain tissue.

Mazahei et al. found downregulation of some chemotaxis-related genes in *Trem2*-deficient microglia and consequently investigated changes in microglial chemotactic activity in the absence of TREM2 expression (Mazaheri et al., 2017). Migration toward injected apoptotic neurons and process extension toward a miniature laser-induced lesion site was impaired in the brains of *Trem2* KO mice, suggesting that TREM2 is required for an appropriate microglial chemotactic response to neuronal injury.

## Concluding remarks

A TREM2/DAP12-mediated signal promotes proliferation, phagocytosis, and migration of microglia by induction and maintenance of microglial activation. However, it remains unclear whether TREM2 is a pro- or anti-inflammatory molecule. Initial studies of microglial TREM2 found that it promoted phagocytosis of apoptotic neurons and suppressed expression of pro-inflammatory molecules such as TNF-α (Takahashi et al., 2005, 2007). Although some studies reported TREM2 as an anti-inflammatory molecule, others proposed a pro-inflammatory role (Table 1). One of the reasons for this controversy may be microglial classification. The microglial phenotype is more complicated than previously thought. Microglia used to be classified into M1 (pro-inflammatory) or M2 (anti-inflammatory) phenotypes according to the expression pattern of marker molecules, as defined in macrophages (Arcuri et al., 2017). However, microglia are distinct from macrophages in terms of their molecular expression profile (Gautier et al., 2012; Hickman et al., 2013; Butovsky et al., 2014). Furthermore, microglia are highly adapted to the CNS environment and have CNS-specific roles (Gosselin et al., 2014; Sierra et al., 2014), suggesting that this simple classification is not appropriate (Mittelbronn, 2014; Ransohoff, 2016). The concepts “disease-associated microglia” or “microglia with neurodegenerative phenotype” have emerged recently (Keren-Shaul et al., 2017; Krasemann et al., 2017) and their molecular expression patterns are related to microglia in the aged brain (Krasemann et al., 2017). Importantly, TREM2 was shown to be the principal inducer of this phenotype, at least in mouse models of AD and ALS (Keren-Shaul et al., 2017; Krasemann et al., 2017). Because the TREM2/DAP12-mediated signal is a dominant switch that transforms microglia from a homeostatic to a disease-associated state, it is likely that dysregulation of TREM2/DAP12 signaling contributes to the pathogenesis of neurodegenerative diseases, including AD. A TREM2/DAP12-targeted strategy could provide new therapies for neurodegenerative diseases.

## Author contributions

All authors listed have made a substantial, direct and intellectual contribution to the work, and approved it for publication.

### Conflict of interest statement

The authors declare that the research was conducted in the absence of any commercial or financial relationships that could be construed as a potential conflict of interest.

## Abbreviations

Aβ: β-amyloid
AD: Alzheimer's disease
ADAM: a disintegrin and metalloproteinase
ALS: amyotrophic lateral sclerosis
ApoE: apolipoprotein E
BDNF: brain-derived neurotrophic factor
CNS: central nervous system
CSF1R: colony-stimulating factor-1 receptor
DAP12: DNAX-activating protein of 12 kDa
EAE: experimental autoimmune encephalomyelitis
ERK: extracellular signal-regulated protein kinase
IFN: interferon
IL: interleukin
ITAM: immunoreceptor tyrosine-based activation
KARAP: killer cell activating receptor-associated protein
KO: knockout
MPTP, 1-methyl-4-phenyl-1, 2, 3: 6-tetrahydropyridine
MPP: 1-methyl-4-phenylpyridinium
mTOR: mammalian target of rapamycin
NO: nitric oxide
NF: nuclear factor
6-OHDA: 6-hydroxydopamine
PD: Parkinson's disease
PI3K: phosphatidylinositol 3-kinase
PLCγ: phospholipase Cγ
PLOSL: polycystic lipomembranous osteodysplasia with sclerosing leukoencephalopathy
ROS: reactive oxygen species
Siglec-H: sialic acid-binding immunoglobulin-like lectin H
SOD1: superoxide dismutase 1
sTREM2: soluble TREM2
TNF: tumor necrosis factor
TREM2: triggering receptor expressed on myeloid cells 2
TYROBP: TYRO protein kinase-binding protein
WT: wild-type.

## References

[B1] Abiega O. Beccari S. Diaz-Aparicio I. Nadjar A. Laye S. Leyrolle Q. (2016). Neuronal hyperactivity disturbs ATP microgradients, impairs microglial motility, and reduces phagocytic receptor expression triggering apoptosis/microglial phagocytosis uncoupling. PLoS Biol. 14:e1002466 10.1371/journal.pbio.1002554 27228556 PMC4881984

[B2] Arcuri C. Mecca C. Bianchi R. Giambanco I. Donato R. (2017). The pathophysiological role of microglia in dynamic surveillance, phagocytosis and structural remodeling of the developing CNS. Front. Mol. Neurosci. 10:191. 10.3389/fnmol.2017.00191 28674485 PMC5474494

[B3] Atagi Y. Liu C. C. Painter M. M. Chen X. F. Verbeeck C. Zheng H.. (2015). Apolipoprotein E Is a ligand for triggering receptor expressed on myeloid cells 2 (TREM2). J. Biol. Chem. 290, 26043–26050. 10.1074/jbc.M115.679043 26374899 PMC4646257

[B4] Bailey C. C. DeVaux L. B. Farzan M. (2015). The triggering receptor expressed on myeloid cells 2 binds apolipoprotein E. J. Biol. Chem. 290, 26033–26042. 10.1074/jbc.M115.677286 26374897 PMC4646256

[B5] Bakker A. B. Hoek R. M. Cerwenka A. Blom B. Lucian L. McNeil T.. (2000a). DAP12-deficient mice fail to develop autoimmunity due to impaired antigen priming. Immunity 13, 345–353. 10.1016/S1074-7613(00)00034-0 11021532

[B6] Bakker A. B. Wu J. Phillips J. H. Lanier L. L. (2000b). NK cell activation: distinct stimulatory pathways counterbalancing inhibitory signals. Hum. Immunol. 61, 18–27. 10.1016/S0198-8859(99)00160-3 10658974

[B7] Belloli S. Pannese M. Buonsanti C. Maiorino C. Di Grigoli G. Carpinelli A.. (2017). Early upregulation of 18-kDa translocator protein in response to acute neurodegenerative damage in TREM2-deficient mice. Neurobiol. Aging 53, 159–168. 10.1016/j.neurobiolaging.2017.01.010 28189343

[B8] Benitez B. A. Cruchaga C. United States-Spain Parkinson's Disease Research G. (2013). TREM2 and neurodegenerative disease. N. Engl. J. Med. 369, 1567–1568. 10.1186/s13024-017-0197-5 24131187 PMC4380008

[B9] Blasius A. L. Cella M. Maldonado J. Takai T. Colonna M. (2006). Siglec-H is an IPC-specific receptor that modulates type I IFN secretion through DAP12. Blood 107, 2474–2476. 10.1182/blood-2005-09-3746 16293595 PMC1895736

[B10] Blasius A. L. Colonna M. (2006). Sampling and signaling in plasmacytoid dendritic cells: the potential roles of Siglec-H. Trends Immunol. 27, 255–260. 10.1016/j.it.2006.04.005 16679063

[B11] Block M. L. Zecca L. Hong J. S. (2007). Microglia-mediated neurotoxicity: uncovering the molecular mechanisms. Nat. Rev. Neurosci. 8, 57–69. 10.1038/nrn2038 17180163

[B12] Bouchon A. Dietrich J. Colonna M. (2000). Cutting edge: inflammatory responses can be triggered by TREM-1, a novel receptor expressed on neutrophils and monocytes. J. Immunol. 164, 4991–4995. 10.4049/jimmunol.164.10.4991 10799849

[B13] Bouchon A. Hernández-Munain C. Cella M. Colonna M. (2001). A DAP12-mediated pathway regulates expression of CC chemokine receptor 7 and maturation of human dendritic cells. J. Exp. Med. 194, 1111–1122. 10.1084/jem.194.8.1111 11602640 PMC2193511

[B14] Brown G. C. Neher J. J. (2014). Microglial phagocytosis of live neurons. Nat. Rev. Neurosci. 15, 209–216. 10.1038/nrn3710 24646669

[B15] Butovsky O. Jedrychowski M. P. Moore C. S. Cialic R. Lanser A. J. Gabriely G.. (2014). Identification of a unique TGF-beta-dependent molecular and functional signature in microglia. Nat. Neurosci. 17, 131–143. 10.1038/nn.3599 24316888 PMC4066672

[B16] Cady J. Koval E. D. Benitez B. A. Zaidman C. Jockel-Balsarotti J. Allred P.. (2014). TREM2 variant p.R47H as a risk factor for sporadic amyotrophic lateral sclerosis. JAMA Neurol. 71, 449–453. 10.1001/jamaneurol.2013.6237 24535663 PMC4087113

[B17] Cambier J. C. (1995). New nomenclature for the Reth motif (or ARH1/TAM/ARAM/YXXL). Immunol. Today 16:110. 10.1016/0167-5699(95)80105-7 7888063

[B18] Cannon J. P. O'Driscoll M. Litman G. W. (2012). Specific lipid recognition is a general feature of CD300 and TREM molecules. Immunogenetics 64, 39–47. 10.1007/s00251-011-0562-4 21800138

[B19] Cantoni C. Bollman B. Licastro D. Xie M. Mikesell R. Schmidt R.. (2015). TREM2 regulates microglial cell activation in response to demyelination *in vivo*. Acta Neuropathol. 129, 429–447. 10.1007/s00401-015-1388-1 25631124 PMC4667728

[B20] Chu F. Shi M. Zheng C. Shen D. Zhu J. Zheng X.. (2018). The roles of macrophages and microglia in multiple sclerosis and experimental autoimmune encephalomyelitis. J. Neuroimmunol. 318, 1–7. 10.1016/j.jneuroim.2018.02.015 29606295

[B21] Colonna M. Wang Y. (2016). TREM2 variants: new keys to decipher Alzheimer disease pathogenesis. Nat. Rev. Neurosci. 17, 201–207. 10.1038/nrn.2016.7 26911435

[B22] Cooper-Knock J. Green C. Altschuler G. Wei W. Bury J. J. Heath P. R.. (2017). A data-driven approach links microglia to pathology and prognosis in amyotrophic lateral sclerosis. Acta Neuropathol. Commun. 5:23. 10.1186/s40478-017-0424-x 28302159 PMC5353945

[B23] Corder E. H. Saunders A. M. Strittmatter W. J. Schmechel D. E. Gaskell P. C. Small G. W.. (1993). Gene dose of apolipoprotein E type 4 allele and the risk of Alzheimer's disease in late onset families. Science 261, 921–923. 10.1126/science.8346443 8346443

[B24] Davalos D. Grutzendler J. Yang G. Kim J. V. Zuo Y. Jung S.. (2005). ATP mediates rapid microglial response to local brain injury *in vivo*. Nat. Neurosci. 8, 752–758. 10.1038/nn1472 15895084

[B25] David S. Kroner A. (2011). Repertoire of microglial and macrophage responses after spinal cord injury. Nat. Rev. Neurosci. 12, 388–399. 10.1038/nrn3053 21673720

[B26] Daws M. R. Lanier L. L. Seaman W. E. Ryan J. C. (2001). Cloning and characterization of a novel mouse myeloid DAP12-associated receptor family. Eur. J. Immunol. 31, 783–791. 10.1002/1521-4141(200103)31:3<783::AID-IMMU783>3.0.CO;2-U 11241283

[B27] Fernández-Arjona M. D. M. Grondona J. M. Granados-Duran P. Fernandez-Llebrez P. Lopez-Avalos M. D. (2017). Microglia morphological categorization in a rat model of neuroinflammation by hierarchical cluster and principal components analysis. Front. Cell. Neurosci. 11:235. 10.3389/fncel.2017.00235 28848398 PMC5550745

[B28] Feuerbach D. Schindler P. Barske C. Joller S. Beng-Louka E. Worringer K. A.. (2017). ADAM17 is the main sheddase for the generation of human triggering receptor expressed in myeloid cells (hTREM2) ectodomain and cleaves TREM2 after Histidine 157. Neurosci. Lett. 660, 109–114. 10.1016/j.neulet.2017.09.034 28923481

[B29] Fu R. Shen Q. Xu P. Luo J. J. Tang Y. (2014). Phagocytosis of microglia in the central nervous system diseases. Mol. Neurobiol. 49, 1422–1434. 10.1007/s12035-013-8620-6 24395130 PMC4012154

[B30] Gautier E. L. Shay T. Miller J. Greter M. Jakubzick C. Ivanov S.. (2012). Gene-expression profiles and transcriptional regulatory pathways that underlie the identity and diversity of mouse tissue macrophages. Nat. Immunol. 13, 1118–1128. 10.1038/ni.2419 23023392 PMC3558276

[B31] Geloso M. C. Corvino V. Marchese E. Serrano A. Michetti F. D'Ambrosi N. (2017). The dual role of microglia in ALS: mechanisms and therapeutic approaches. Front. Aging Neurosci. 9:242. 10.3389/fnagi.2017.00242 28790913 PMC5524666

[B32] Gosselin D. Link V. M. Romanoski C. E. Fonseca G. J. Eichenfield D. Z. Spann N. J.. (2014). Environment drives selection and function of enhancers controlling tissue-specific macrophage identities. Cell 159, 1327–1340. 10.1016/j.cell.2014.11.023 25480297 PMC4364385

[B33] Graeber M. B. Streit W. J. Kreutzberg G. W. (1988). Axotomy of the rat facial nerve leads to increased CR3 complement receptor expression by activated microglial cells. J. Neurosci. Res. 21, 18–24. 10.1002/jnr.490210104 3216409

[B34] Guan Z. Kuhn J. A. Wang X. Colquitt B. Solorzano C. Vaman S.. (2016). Injured sensory neuron-derived CSF1 induces microglial proliferation and DAP12-dependent pain. Nat. Neurosci. 19, 94–101. 10.1038/nn.4189 26642091 PMC4703328

[B35] Guerreiro R. Wojtas A. Bras J. Carrasquillo M. Rogaeva E. Majounie E.. (2013). TREM2 variants in Alzheimer's disease. N. Engl. J. Med. 368, 117–127. 10.1056/NEJMoa1211851 23150934 PMC3631573

[B36] Guruswamy R. ElAli A. (2017). Complex roles of microglial cells in ischemic stroke pathobiology: new insights and future directions. Int. J. Mol. Sci. 18:E496. 10.3390/ijms18030496 28245599 PMC5372512

[B37] Hamerman J. A. Jarjoura J. R. Humphrey M. B. Nakamura M. C. Seaman W. E. Lanier L. L. (2006). Cutting edge: inhibition of TLR and FcR responses in macrophages by triggering receptor expressed on myeloid cells (TREM)-2 and DAP12. J. Immunol. 177, 2051–2055. 10.4049/jimmunol.177.4.2051 16887962

[B38] Hanisch U. K. Kettenmann H. (2007). Microglia: active sensor and versatile effector cells in the normal and pathologic brain. Nat. Neurosci. 10, 1387–1394. 10.1038/nn1997 17965659

[B39] Heslegrave A. Heywood W. Paterson R. Magdalinou N. Svensson J. Johansson P.. (2016). Increased cerebrospinal fluid soluble TREM2 concentration in Alzheimer's disease. Mol. Neurodegener. 11:3. 10.1186/s13024-016-0071-x 26754172 PMC4709982

[B40] Hickman S. E. Kingery N. D. Ohsumi T. K. Borowsky M. L. Wang L. C. Means T. K.. (2013). The microglial sensome revealed by direct RNA sequencing. Nat. Neurosci. 16, 1896–1905. 10.1038/nn.3554 24162652 PMC3840123

[B41] Hu X. Leak R. K. Shi Y. Suenaga J. Gao Y. Zheng P.. (2015). Microglial and macrophage polarization-new prospects for brain repair. Nat. Rev. Neurol. 11, 56–64. 10.1038/nrneurol.2014.207 25385337 PMC4395497

[B42] Hu X. Zhang D. Pang H. Caudle W. M. Li Y. Gao H.. (2008). Macrophage antigen complex-1 mediates reactive microgliosis and progressive dopaminergic neurodegeneration in the MPTP model of Parkinson's disease. J. Immunol. 181, 7194–7204. 10.4049/jimmunol.181.10.7194 18981141 PMC2759089

[B43] Inoue K. Tsuda M. (2018). Microglia in neuropathic pain: cellular and molecular mechanisms and therapeutic potential. Nat. Rev. Neurosci. 19, 138–152. 10.1038/nrn.2018.2 29416128

[B44] Ito H. Hamerman J. A. (2012). TREM-2, triggering receptor expressed on myeloid cell-2, negatively regulates TLR responses in dendritic cells. Eur. J. Immunol. 42, 176–185. 10.1002/eji.201141679 21956652 PMC3444819

[B45] Jay T. R. Hirsch A. M. Broihier M. L. Miller C. M. Neilson L. E. Ransohoff R. M.. (2017). Disease progression-dependent effects of TREM2 deficiency in a mouse model of Alzheimer's disease. J. Neurosci. 37, 637–647. 10.1523/JNEUROSCI.2110-16.2016 28100745 PMC5242410

[B46] Jay T. R. Miller C. M. Cheng P. J. Graham L. C. Bemiller S. Broihier M. L.. (2015). TREM2 deficiency eliminates TREM2^+^ inflammatory macrophages and ameliorates pathology in Alzheimer's disease mouse models. J. Exp. Med. 212, 287–295. 10.1084/jem.20142322 25732305 PMC4354365

[B47] Jonsson T. Stefansson H. Steinberg S. Jonsdottir I. Jonsson P. V. Snaedal J.. (2013). Variant of TREM2 associated with the risk of Alzheimer's disease. N. Engl. J. Med. 368, 107–116. 10.1056/NEJMoa1211103 23150908 PMC3677583

[B48] Jonsson T. Stefansson K. (2013). TREM2 and neurodegenerative disease. N. Engl. J. Med. 369, 1568–1569. 10.1016/j.molmed.2017.03.008 24131183

[B49] Kaifu T. Nakahara J. Inui M. Mishima K. Momiyama T. Kaji M.. (2003). Osteopetrosis and thalamic hypomyelinosis with synaptic degeneration in DAP12-deficient mice. J. Clin. Invest. 111, 323–332. 10.1172/JCI16923 12569157 PMC151867

[B50] Kawabori M. Kacimi R. Kauppinen T. Calosing C. Kim J. Y. Hsieh C. L.. (2015). Triggering receptor expressed on myeloid cells 2 (TREM2) deficiency attenuates phagocytic activities of microglia and exacerbates ischemic damage in experimental stroke. J. Neurosci. 35, 3384–3396. 10.1523/JNEUROSCI.2620-14.2015 25716838 PMC4339351

[B51] Keren-Shaul H. Spinrad A. Weiner A. Matcovitch-Natan O. Dvir-Szternfeld R. Ulland T. K.. (2017). A unique microglia type associated with restricting development of alzheimer's disease. Cell 169, 1276.e17–1290.e17. 10.1016/j.cell.2017.05.018 28602351

[B52] Kierdorf K. Prinz M. (2013). Factors regulating microglia activation. Front. Cell. Neurosci. 7:44. 10.3389/fncel.2013.00044 23630462 PMC3632747

[B53] Kinugawa K. Monnet Y. Bechade C. Alvarez-Fischer D. Hirsch E. C. Bessis A. (2013). DAP12 and CD11b contribute to the microglial-induced death of dopaminergic neurons *in vitro* but not *in vivo* in the MPTP mouse model of Parkinson's disease. J. Neuroinflammation 10:82 10.1186/1742-2094-10-82 23844828 PMC3720270

[B54] Kleinberger G. Yamanishi Y. Suárez-Calvet M. Czirr E. Lohmann E. Cuyvers E.. (2014). TREM2 mutations implicated in neurodegeneration impair cell surface transport and phagocytosis. Sci. Transl. Med. 6:243ra286. 10.1126/scitranslmed.3009093 24990881

[B55] Kobayashi K. Kobayashi E. Miyazu K. Muramori F. Hiramatsu S. Aoki T.. (2000). Hypothalamic haemorrhage and thalamus degeneration in a case of Nasu-Hakola disease with hallucinatory symptoms and central hypothermia. Neuropathol. Appl. Neurobiol. 26, 98–101. 10.1046/j.1365-2990.2000.00224.x 10787346

[B56] Kobayashi M. Konishi H. Sayo A. Takai T. Kiyama H. (2016). TREM2/DAP12 signal elicits proinflammatory response in microglia and exacerbates neuropathic pain. J. Neurosci. 36, 11138–11150. 10.1523/JNEUROSCI.1238-16.2016 27798193 PMC6705657

[B57] Kobayashi M. Konishi H. Takai T. Kiyama H. (2015). A DAP12-dependent signal promotes pro-inflammatory polarization in microglia following nerve injury and exacerbates degeneration of injured neurons. Glia 63, 1073–1082. 10.1002/glia.22802 25690660

[B58] Kober D. L. Brett T. J. (2017). TREM2-ligand interactions in health and disease. J. Mol. Biol. 429, 1607–1629. 10.1016/j.jmb.2017.04.004 28432014 PMC5485854

[B59] Konishi H. Kobayashi M. Kunisawa T. Imai K. Sayo A. Malissen B.. (2017). Siglec-H is a microglia-specific marker that discriminates microglia from CNS-associated macrophages and CNS-infiltrating monocytes. Glia 65, 1927–1943. 10.1002/glia.23204 28836308

[B60] Krasemann S. Madore C. Cialic R. Baufeld C. Calcagno N. El Fatimy R.. (2017). The TREM2-APOE pathway drives the transcriptional phenotype of dysfunctional microglia in neurodegenerative diseases. Immunity 47, 566.e9–581.e9. 10.1016/j.immuni.2017.08.008 28930663 PMC5719893

[B61] Lampron A. Larochelle A. Laflamme N. Préfontaine P. Plante M. M. Sánchez M. G.. (2015). Inefficient clearance of myelin debris by microglia impairs remyelinating processes. J. Exp. Med. 212, 481–495. 10.1084/jem.20141656 25779633 PMC4387282

[B62] Lanier L. L. (2009). DAP10- and DAP12-associated receptors in innate immunity. Immunol. Rev. 227, 150–160. 10.1111/j.1600-065X.2008.00720.x 19120482 PMC2794881

[B63] Lanier L. L. Corliss B. C. Wu J. Leong C. Phillips J. H. (1998). Immunoreceptor DAP12 bearing a tyrosine-based activation motif is involved in activating NK cells. Nature 391, 703–707. 10.1038/35642 9490415

[B64] Lee C. Y. D. Daggett A. Gu X. Jiang L. L. Langfelder P. Li X.. (2018). Elevated TREM2 gene dosage reprograms microglia responsivity and ameliorates pathological phenotypes in Alzheimer's disease models. Neuron 97, 1032.e5–1048.e5. 10.1016/j.neuron.2018.02.002 29518357 PMC5927822

[B65] Lill C. M. Rengmark A. Pihlstrøm L. Fogh I. Shatunov A. Sleiman P. M.. (2015). The role of TREM2 R47H as a risk factor for Alzheimer's disease, frontotemporal lobar degeneration, amyotrophic lateral sclerosis, and Parkinson's disease. Alzheimers Dement. 11, 1407–1416. 10.1016/j.jalz.2014.12.009 25936935 PMC4627856

[B66] Linnartz-Gerlach B. Kopatz J. Neumann H. (2014). Siglec functions of microglia. Glycobiology 24, 794–799. 10.1093/glycob/cwu044 24833613

[B67] Liu G. Liu Y. Jiang Q. Jiang Y. Feng R. Zhang L.. (2016). Convergent genetic and expression datasets highlight TREM2 in Parkinson's disease susceptibility. Mol. Neurobiol. 53, 4931–4938. 10.1007/s12035-015-9416-7 26365049

[B68] Liu J. Wang F. (2017). Role of neuroinflammation in amyotrophic lateral sclerosis: cellular mechanisms and therapeutic implications. Front. Immunol. 8:1005. 10.3389/fimmu.2017.01005 28871262 PMC5567007

[B69] Lobsiger C. S. Boillee S. Pozniak C. Khan A. M. McAlonis-Downes M. Lewcock J. W. (2013). C1q induction and global complement pathway activation do not contribute to ALS toxicity in mutant SOD1 mice. Proc. Natl. Acad. Sci. U.S.A. 110, E4385–E4392. 10.1073/pnas.1318309110 24170856 PMC3831990

[B70] Ma J. Jiang T. Tan L. Yu J. T. (2015). TYROBP in Alzheimer's disease. Mol. Neurobiol. 51, 820–826. 10.1007/s12035-014-8811-9 25052481

[B71] Ma Y. Wang J. Wang Y. Yang G. Y. (2017). The biphasic function of microglia in ischemic stroke. Prog. Neurobiol. 157, 247–272. 10.1016/j.pneurobio.2016.01.005 26851161

[B72] Marta M. (2009). Toll-like receptors in multiple sclerosis mouse experimental models. Ann. N. Y. Acad. Sci. 1173, 458–462. 10.1111/j.1749-6632.2009.04849.x 19758186

[B73] Mazaheri F. Snaidero N. Kleinberger G. Madore C. Daria A. Werner G.. (2017). TREM2 deficiency impairs chemotaxis and microglial responses to neuronal injury. EMBO Rep. 18, 1186–1198. 10.15252/embr.201743922 28483841 PMC5494532

[B74] Mecca C. Giambanco I. Donato R. Arcuri C. (2018). Microglia and aging: the role of the TREM2-DAP12 and CX3CL1-CX3CR1 axes. Int. J. Mol. Sci. 19:E318. 10.3390/ijms19010318 29361745 PMC5796261

[B75] Miranda-Hernandez S. Baxter A. G. (2013). Role of toll-like receptors in multiple sclerosis. Am. J. Clin. Exp. Immunol. 2, 75–93. 23885326 PMC3714200

[B76] Miron V. E. Boyd A. Zhao J. W. Yuen T. J. Ruckh J. M. Shadrach J. L.. (2013). M2 microglia and macrophages drive oligodendrocyte differentiation during CNS remyelination. Nat. Neurosci. 16, 1211–1218. 10.1038/nn.3469 23872599 PMC3977045

[B77] Mittelbronn M. (2014). The M1/M2 immune polarization concept in microglia: a fair transfer. Neuroimmunol. Neuroinflamm 1, 6–7. 10.4103/2347-8659.135567

[B78] Mohammad M. G. Hassanpour M. Tsai V. W. Li H. Ruitenberg M. J. Booth D. W.. (2012). Dendritic cells and multiple sclerosis: disease, tolerance and therapy. Int. J. Mol. Sci. 14, 547–562. 10.3390/ijms14010547 23271370 PMC3565281

[B79] Nakajima K. Kohsaka S. (2004). Microglia: neuroprotective and neurotrophic cells in the central nervous system. Curr. Drug Targets Cardiovasc. Haematol. Disord. 4, 65–84. 10.2174/1568006043481284 15032653

[B80] Nataf S. Anginot A. Vuaillat C. Malaval L. Fodil N. Chereul E.. (2005). Brain and bone damage in KARAP/DAP12 loss-of-function mice correlate with alterations in microglia and osteoclast lineages. Am. J. Pathol. 166, 275–286. 10.1016/S0002-9440(10)62251-1 15632019 PMC1602283

[B81] Neumann H. Kotter M. R. Franklin R. J. (2009). Debris clearance by microglia: an essential link between degeneration and regeneration. Brain 132(Pt 2), 288–295. 10.1093/brain/awn109 18567623 PMC2640215

[B82] Nimmerjahn A. Kirchhoff F. Helmchen F. (2005). Resting microglial cells are highly dynamic surveillants of brain parenchyma *in vivo*. Science 308, 1314–1318. 10.1126/science.1110647 15831717

[B83] Otero K. Turnbull I. R. Poliani P. L. Vermi W. Cerutti E. Aoshi T.. (2009). Macrophage colony-stimulating factor induces the proliferation and survival of macrophages via a pathway involving DAP12 and beta-catenin. Nat. Immunol. 10, 734–743. 10.1038/ni.1744 19503107 PMC4004764

[B84] Paloneva J. Autti T. Raininko R. Partanen J. Salonen O. Puranen M.. (2001). CNS manifestations of Nasu-Hakola disease: a frontal dementia with bone cysts. Neurology 56, 1552–1558. 10.1212/WNL.56.11.1552 11402114

[B85] Paloneva J. Kestilä M. Wu J. Salminen A. Böhling T. Ruotsalainen V.. (2000). Loss-of-function mutations in TYROBP (DAP12) result in a presenile dementia with bone cysts. Nat. Genet. 25, 357–361. 10.1038/77153 10888890

[B86] Paloneva J. Manninen T. Christman G. Hovanes K. Mandelin J. Adolfsson R.. (2002). Mutations in two genes encoding different subunits of a receptor signaling complex result in an identical disease phenotype. Am. J. Hum. Genet. 71, 656–662. 10.1086/342259 12080485 PMC379202

[B87] Paolicelli R. C. Bolasco G. Pagani F. Maggi L. Scianni M. Panzanelli P.. (2011). Synaptic pruning by microglia is necessary for normal brain development. Science 333, 1456–1458. 10.1126/science.1202529 21778362

[B88] Parkhurst C. N. Yang G. Ninan I. Savas J. N. Yates J. R. III Lafaille J. J.. (2013). Microglia promote learning-dependent synapse formation through brain-derived neurotrophic factor. Cell 155, 1596–1609. 10.1016/j.cell.2013.11.030 24360280 PMC4033691

[B89] Peng Q. Malhotra S. Torchia J. A. Kerr W. G. Coggeshall K. M. Humphrey M. B. (2010). TREM2- and DAP12-dependent activation of PI3K requires DAP10 and is inhibited by SHIP1. Sci. Signal. 3:ra38. 10.1126/scisignal.2000500 20484116 PMC2900152

[B90] Piccio L. Buonsanti C. Cella M. Tassi I. Schmidt R. E. Fenoglio C.. (2008). Identification of soluble TREM-2 in the cerebrospinal fluid and its association with multiple sclerosis and CNS inflammation. Brain 131(Pt 11), 3081–3091. 10.1093/brain/awn217 18790823 PMC2577803

[B91] Piccio L. Buonsanti C. Mariani M. Cella M. Gilfillan S. Cross A. H.. (2007). Blockade of TREM-2 exacerbates experimental autoimmune encephalomyelitis. Eur. J. Immunol. 37, 1290–1301. 10.1002/eji.200636837 17407101

[B92] Piccio L. Deming Y. Del-Águila J. L. Ghezzi L. Holtzman D. M. Fagan A. M.. (2016). Cerebrospinal fluid soluble TREM2 is higher in Alzheimer disease and associated with mutation status. Acta Neuropathol. 131, 925–933. 10.1007/s00401-016-1533-5 26754641 PMC4867123

[B93] Poliani P. L. Wang Y. Fontana E. Robinette M. L. Yamanishi Y. Gilfillan S.. (2015). TREM2 sustains microglial expansion during aging and response to demyelination. J. Clin. Invest. 125, 2161–2170. 10.1172/JCI77983 25893602 PMC4463196

[B94] Ransohoff R. M. (2016). A polarizing question: do M1 and M2 microglia exist? Nat. Neurosci. 19, 987–991. 10.1038/nn.4338 27459405

[B95] Rayaprolu S. Mullen B. Baker M. Lynch T. Finger E. Seeley W. W.. (2013). TREM2 in neurodegeneration: evidence for association of the p.R47H variant with frontotemporal dementia and Parkinson's disease. Mol. Neurodegener. 8:19. 10.1186/1750-1326-8-19 23800361 PMC3691612

[B96] Ren M. Guo Y. Wei X. Yan S. Qin Y. Zhang X.. (2018). TREM2 overexpression attenuates neuroinflammation and protects dopaminergic neurons in experimental models of Parkinson's disease. Exp. Neurol. 302, 205–213. 10.1016/j.expneurol.2018.01.016 29407460

[B97] Roth T. L. Nayak D. Atanasijevic T. Koretsky A. P. Latour L. L. McGavern D. B. (2014). Transcranial amelioration of inflammation and cell death after brain injury. Nature 505, 223–228. 10.1038/nature12808 24317693 PMC3930079

[B98] Roumier A. Béchade C. Poncer J. C. Smalla K. H. Tomasello E. Vivier E.. (2004). Impaired synaptic function in the microglial KARAP/DAP12-deficient mouse. J. Neurosci. 24, 11421–11428. 10.1523/JNEUROSCI.2251-04.2004 15601948 PMC6730361

[B99] Saber M. Kokiko-Cochran O. Puntambekar S. S. Lathia J. D. Lamb B. T. (2017). Triggering receptor expressed on myeloid cells 2 deficiency alters acute macrophage distribution and improves recovery after traumatic brain injury. J. Neurotrauma 34, 423–435. 10.1089/neu.2016.4401 26976047

[B100] Schlepckow K. Kleinberger G. Fukumori A. Feederle R. Lichtenthaler S. F. Steiner H.. (2017). An Alzheimer-associated TREM2 variant occurs at the ADAM cleavage site and affects shedding and phagocytic function. EMBO Mol. Med. 9, 1356–1365. 10.15252/emmm.201707672 28855300 PMC5623859

[B101] Schmid C. D. Sautkulis L. N. Danielson P. E. Cooper J. Hasel K. W. Hilbush B. S.. (2002). Heterogeneous expression of the triggering receptor expressed on myeloid cells-2 on adult murine microglia. J. Neurochem. 83, 1309–1320. 10.1046/j.1471-4159.2002.01243.x 12472885 PMC2637869

[B102] Sieber M. W. Jaenisch N. Brehm M. Guenther M. Linnartz-Gerlach B. Neumann H.. (2013). Attenuated inflammatory response in triggering receptor expressed on myeloid cells 2 (TREM2) knock-out mice following stroke. PLoS ONE 8:e52982. 10.1371/journal.pone.0052982 23301011 PMC3536811

[B103] Sierra A. Tremblay M. È. Wake H. (2014). Never-resting microglia: physiological roles in the healthy brain and pathological implications. Front. Cell. Neurosci. 8:240. 10.3389/fncel.2014.00240 25177273 PMC4133768

[B104] Suárez-Calvet M. Kleinberger G. Araque Caballero M. Á. Brendel M. Rominger A. Alcolea D.. (2016). sTREM2 cerebrospinal fluid levels are a potential biomarker for microglia activity in early-stage Alzheimer's disease and associate with neuronal injury markers. EMBO Mol. Med. 8, 466–476. 10.15252/emmm.201506123 26941262 PMC5120370

[B105] Subramaniam S. R. Federoff H. J. (2017). Targeting microglial activation states as a therapeutic avenue in Parkinson's disease. Front. Aging Neurosci. 9:176. 10.3389/fnagi.2017.00176 28642697 PMC5463358

[B106] Takagi H. Arimura K. Uto T. Fukaya T. Nakamura T. Choijookhuu N.. (2016). Plasmacytoid dendritic cells orchestrate TLR7-mediated innate and adaptive immunity for the initiation of autoimmune inflammation. Sci. Rep. 6:24477. 10.1038/srep24477 27075414 PMC4830934

[B107] Takagi H. Fukaya T. Eizumi K. Sato Y. Sato K. Shibazaki A.. (2011). Plasmacytoid dendritic cells are crucial for the initiation of inflammation and T cell immunity *in vivo*. Immunity 35, 958–971. 10.1016/j.immuni.2011.10.014 22177923

[B108] Takahashi K. Prinz M. Stagi M. Chechneva O. Neumann H. (2007). TREM2-transduced myeloid precursors mediate nervous tissue debris clearance and facilitate recovery in an animal model of multiple sclerosis. PLoS Med. 4:e124. 10.1371/journal.pmed.0040124 17425404 PMC1851623

[B109] Takahashi K. Rochford C. D. Neumann H. (2005). Clearance of apoptotic neurons without inflammation by microglial triggering receptor expressed on myeloid cells-2. J. Exp. Med. 201, 647–657. 10.1084/jem.20041611 15728241 PMC2213053

[B110] Thrash J. C. Torbett B. E. Carson M. J. (2009). Developmental regulation of TREM2 and DAP12 expression in the murine CNS: implications for Nasu-Hakola disease. Neurochem. Res. 34, 38–45. 10.1007/s11064-008-9657-1 18404378 PMC2655126

[B111] Tomasello E. Desmoulins P. O. Chemin K. Guia S. Cremer H. Ortaldo J.. (2000). Combined natural killer cell and dendritic cell functional deficiency in KARAP/DAP12 loss-of-function mutant mice. Immunity 13, 355–364. 10.1016/S1074-7613(00)00035-2 11021533

[B112] Turnbull I. R. Colonna M. (2007). Activating and inhibitory functions of DAP12. Nat. Rev. Immunol. 7, 155–161. 10.1038/nri2014 17220916

[B113] Turnbull I. R. Gilfillan S. Cella M. Aoshi T. Miller M. Piccio L.. (2006). Cutting edge: TREM-2 attenuates macrophage activation. J. Immunol. 177, 3520–3524. 10.4049/jimmunol.177.6.3520 16951310

[B114] Ueno M. Fujita Y. Tanaka T. Nakamura Y. Kikuta J. Ishii M.. (2013). Layer V cortical neurons require microglial support for survival during postnatal development. Nat. Neurosci. 16, 543–551. 10.1038/nn.3358 23525041

[B115] Ulland T. K. Song W. M. Huang S. C. Ulrich J. D. Sergushichev A. Beatty W. L.. (2017). TREM2 maintains microglial metabolic fitness in Alzheimer's disease. Cell 170, 649.e13–663.e13. 10.1016/j.cell.2017.07.023 28802038 PMC5573224

[B116] Verloes A. Maquet P. Sadzot B. Vivario M. Thiry A. Franck G. (1997). Nasu-Hakola syndrome: polycystic lipomembranous osteodysplasia with sclerosing leucoencephalopathy and presenile dementia. J. Med. Genet. 34, 753–757. 10.1136/jmg.34.9.753 9321763 PMC1051061

[B117] Virgone-Carlotta A. Uhlrich J. Akram M. N. Ressnikoff D. Chretien F. Domenget C.. (2013). Mapping and kinetics of microglia/neuron cell-to-cell contacts in the 6-OHDA murine model of Parkinson's disease. Glia 61, 1645–1658. 10.1002/glia.22546 23893349

[B118] Wake H. Moorhouse A. J. Jinno S. Kohsaka S. Nabekura J. (2009). Resting microglia directly monitor the functional state of synapses *in vivo* and determine the fate of ischemic terminals. J. Neurosci. 29, 3974–3980. 10.1523/JNEUROSCI.4363-08.2009 19339593 PMC6665392

[B119] Wakselman S. Béchade C. Roumier A. Bernard D. Triller A. Bessis A. (2008). Developmental neuronal death in hippocampus requires the microglial CD11b integrin and DAP12 immunoreceptor. J. Neurosci. 28, 8138–8143. 10.1523/JNEUROSCI.1006-08.2008 18685038 PMC6670768

[B120] Wang Y. Cella M. Mallinson K. Ulrich J. D. Young K. L. Robinette M. L.. (2015). TREM2 lipid sensing sustains the microglial response in an Alzheimer's disease model. Cell 160, 1061–1071. 10.1016/j.cell.2015.01.049 25728668 PMC4477963

[B121] Wang Y. Ulland T. K. Ulrich J. D. Song W. Tzaferis J. A. Hole J. T.. (2016). TREM2-mediated early microglial response limits diffusion and toxicity of amyloid plaques. J. Exp. Med. 213, 667–675. 10.1084/jem.20151948 27091843 PMC4854736

[B122] Wlodarczyk A. Holtman I. R. Krueger M. Yogev N. Bruttger J. Khorooshi R.. (2017). A novel microglial subset plays a key role in myelinogenesis in developing brain. EMBO J. 36, 3292–3308. 10.15252/embj.201696056 28963396 PMC5686552

[B123] Wu D. C. Teismann P. Tieu K. Vila M. Jackson-Lewis V. Ischiropoulos H.. (2003). NADPH oxidase mediates oxidative stress in the 1-methyl-4-phenyl-1,2,3,6-tetrahydropyridine model of Parkinson's disease. Proc. Natl. Acad. Sci. U.S.A. 100, 6145–6150. 10.1073/pnas.0937239100 12721370 PMC156340

[B124] Wu R. Li X. Xu P. Huang L. Cheng J. Huang X.. (2017). TREM2 protects against cerebral ischemia/reperfusion injury. Mol. Brain 10:20. 10.1186/s13041-017-0296-9 28592261 PMC5461720

[B125] Wyatt S. K. Witt T. Barbaro N. M. Cohen-Gadol A. A. Brewster A. L. (2017). Enhanced classical complement pathway activation and altered phagocytosis signaling molecules in human epilepsy. Exp. Neurol. 295, 184–193. 10.1016/j.expneurol.2017.06.009 28601603

[B126] Yamada J. Jinno S. (2011). Alterations in neuronal survival and glial reactions after axotomy by ceftriaxone and minocycline in the mouse hypoglossal nucleus. Neurosci. Lett. 504, 295–300. 10.1016/j.neulet.2011.09.051 21970974

[B127] Yeh F. L. Wang Y. Tom I. Gonzalez L. C. Sheng M. (2016). TREM2 binds to apolipoproteins, including APOE and CLU/APOJ, and thereby facilitates uptake of amyloid-beta by microglia. Neuron 91, 328–340. 10.1016/j.neuron.2016.06.015 27477018

[B128] Yuan P. Condello C. Keene C. D. Wang Y. Bird T. D. Paul S. M.. (2016). TREM2 haplodeficiency in mice and humans impairs the microglia barrier function leading to decreased amyloid compaction and severe axonal dystrophy. Neuron 92, 252–264. 10.1016/j.neuron.2016.09.016 27710785

[B129] Zhai Q. Li F. Chen X. Jia J. Sun S. Zhou D.. (2017). Triggering receptor expressed on myeloid cells 2, a novel regulator of immunocyte phenotypes, confers neuroprotection by relieving neuroinflammation. Anesthesiology 127, 98–110. 10.1097/ALN.0000000000001628 28398927

[B130] Zhang B. Gaiteri C. Bodea L. G. Wang Z. McElwee J. Podtelezhnikov A. A.. (2013). Integrated systems approach identifies genetic nodes and networks in late-onset Alzheimer's disease. Cell 153, 707–720. 10.1016/j.cell.2013.03.030 23622250 PMC3677161

[B131] Zhao Y. Wu X. Li X. Jiang L. L. Gui X. Liu Y.. (2018). TREM2 is a receptor for beta-amyloid that mediates microglial function. Neuron 97, 1023.e7–1031.e7. 10.1016/j.neuron.2018.01.031 29518356 PMC5889092

[B132] Zheng H. Jia L. Liu C. C. Rong Z. Zhong L. Yang L.. (2017). TREM2 promotes microglial survival by activating Wnt/beta-catenin pathway. J. Neurosci. 37, 1772–1784. 10.1523/JNEUROSCI.2459-16.2017 28077724 PMC5320608

[B133] Zheng X. Y. Zhang H. L. Luo Q. Zhu J. (2011). Kainic acid-induced neurodegenerative model: potentials and limitations. J. Biomed. Biotechnol. 2011:457079. 10.1155/2011/457079 21127706 PMC2992819

[B134] Zhong L. Chen X. F. Wang T. Wang Z. Liao C. Wang Z.. (2017a). Soluble TREM2 induces inflammatory responses and enhances microglial survival. J. Exp. Med. 214, 597–607. 10.1084/jem.20160844 28209725 PMC5339672

[B135] Zhong L. Zhang Z. L. Li X. Liao C. Mou P. Wang T.. (2017b). TREM2/DAP12 complex regulates inflammatory responses in microglia via the JNK signaling pathway. Front. Aging Neurosci. 9:204. 10.3389/fnagi.2017.00204 28680398 PMC5478682

[B136] Ziebell J. M. Morganti-Kossmann M. C. (2010). Involvement of pro- and anti-inflammatory cytokines and chemokines in the pathophysiology of traumatic brain injury. Neurotherapeutics 7, 22–30. 10.1016/j.nurt.2009.10.016 20129494 PMC5084109

[B137] Zou W. Reeve J. L. Liu Y. Teitelbaum S. L. Ross F. P. (2008). DAP12 couples c-Fms activation to the osteoclast cytoskeleton by recruitment of Syk. Mol. Cell 31, 422–431. 10.1016/j.molcel.2008.06.023 18691974 PMC2584874

